# Supplementary material for: Criticality supports cross-frequency cortical-thalamic information transfer during conscious states
Source: eLife. 2024 Jan 5;13:e86547. doi: 10.7554/eLife.86547 (PMC10805384; doi:10.7554/eLife.86547)
Supplement: Supplementary file 1. [file elife-86547-supp1.pdf]

This table presents a side-by-side comparison of the results obtained from our cross-frequency information transfer analysis using 100 and 250 surrogate datasets. Each row corresponds to a selected 10-second trial during the conscious state for each patient/animal. The selection criteria for these trials were as follows: we identified a trial in which the initial analysis (using 100 surrogates) showed statistically significant cross-frequency information transfer in both directions, i.e. from both cortex ("cort") to thalamus ("thal") and from thalamus to cortex. In a majority of subjects, this occurred for a large portion of available trials, and so we randomly selected among those a single trial to evaluate with the larger number of surrogates. In instances where no trials showed significant bidirectional communication, we selected the trial which had the lowest combined p-value (sum of the p-value for cortico-thalamic communication and the p-value for thalamo-cortical communication). The table illustrates that our conclusions hold consistent when the number of surrogates was increased to 250, thereby reinforcing the robustness of our original findings.

	100 Surrogates		250 Surrogates	
	Cort to thal	Thal to cort	Cort to thal	Thal to cort
Human ET Patient 1	p=0	p=0	p=0	p=0
Human ET Patient 2	p=0	p=0	p=0	p=0
Human ET Patient 3	p=0	p=0	p=0	p=0
Human ET Patient 4	p=0	p=0	p=0	p=0
Human ET Patient 5	p=0	p=0	p=0	p=0
Human ET Patient 6	p=0	p=0	p=0	p=0
Human ET Patient 7	p=0	p=0	p=0	p=0
Human ET Patient 8	p=0	p=0	p=0	p=0
Human ET Patient 9	p=0	p=0	p=0	p=0
Human ET Patient 10	p=0	p=0	p=0	p=0
Long-Evans Rat 1	p=0.01	p=0.04	p=0.016	p=0.02
Long-Evans Rat 2	p=0.03	p=0.04	p=0.028	p=0.036
Long-Evans Rat 3	p=0.01	p=0.02	p=0.036	p=0.004
Long-Evans Rat 4	p=0	p=0.02	p=0	p=0.004
Long-Evans Rat 5	p=0.02	p=0.02	p=0.02	p=0.016
Long-Evans Rat 6	p=0.03	p=0.02	p=0.012	p=0.024
Long-Evans Rat 7	p=0.14	p=0.03	p=0.136	p=0.004
Long-Evans Rat 8	p=0.01	p=0.024	p=0.04	p=0.04
Long-Evans Rat 9	p=0.07	p=0.16	p=0.04	p=0.14
GAERS Rat 1	p=0	p=0.02	p=0.004	p=0.004
GAERS Rat 2	p=0.03	p=0	p=0.052	p=0.008
GAERS Rat 3	p=0.01	p=0	p=0.004	p=0
GAERS Rat 4	p=0	p=0	p=0	p=0.01
GAERS Rat 5	p=0.01	p=0.03	p=0.012	p=0.028
GAERS Rat 6	p=0	p=0.03	p=0.008	p=0.044
GAERS Rat 7	p=0	p=0.01	p=0.008	p=0.028
C58/BL6 Mouse 1	p=0.03	p=0.01	p=0.008	p=0.012
C58/BL6 Mouse 2	p=0.02	p=0	p=0.008	p=0
C58/BL6 Mouse 3	p=0	p=0.03	p=0.004	p=0.008
C58/BL6 Mouse 4	p=0	p=0	p=0.012	p=0.004
C58/BL6 Mouse 5	p=0.02	p=0	p=0.036	p=0.02
