# Supplementary material for: Criticality supports cross-frequency cortical-thalamic information transfer during conscious states
Source: eLife. 2024 Jan 5;13:e86547. doi: 10.7554/eLife.86547 (PMC10805384; doi:10.7554/eLife.86547)
Supplement: Supplementary file 2. [file elife-86547-supp2.pdf]

Table 1: We tested whether the variance of cross-frequency corticothalamic information transfer across brain states was potentially driven by changes in the spectral power of slow (1-13 Hz) or fast (52-104 Hz) thalamocortical activity. To do so, we used a permutation-based nonparametric ANCOVA with 10,000 permutations. We set the response variable as the median strength of cross-frequency corticothalamic information transfer for each brain state in each subject, and set the group label as brain state (i.e. normal waking, generalized spike-and-wave seizure, anesthesia, or 5-MeO-DMT). We set the covariates to the median relative spectral power between 1 and 13 Hz across all cortical and thalamic channels and the median relative spectral power between 52 and 104 Hz across all cortical and thalamic channels. We found that only brain state significantly explained the observed variance across subjects in the strength of cross-frequency corticothalamic information transfer, with no significant effect of spectral power or its interactions with brain state.

Variable	F-statistic	p-value
Brain state	95.1718	0.0001
Thalamocortical low-frequency power	0.0014	0.9337
Thalamocortical high-frequency power	0.54501	0.4714
Brain state*low-frequency power	20.3632	0.0772
Brain state*high-frequency power	18.8499	0.0903

Table 2: We tested whether the variance of cross-frequency information transfer from thalamus to cortex across brain states was potentially driven by changes in the relative spectral power of slow (1-13 Hz) or fast (52-104 Hz) thalamocortical activity, following the same method as in Table 1. We again found that only brain state significantly explained the observed variance across subjects in the strength of cross-frequency thalamocortical information transfer, with no significant effect of spectral power or its interactions with brain state.

Variable	F-statistic	p-value
Brain state	79.0631	0.0001
Thalamocortical low-frequency power	1.2742	0.21
Thalamocortical high-frequency power	3.987	0.0521
Brain state*low-frequency power	4.7073	0.0968
Brain state*high-frequency power	5.1115	0.0835
