# Supplementary material for: Criticality supports cross-frequency cortical-thalamic information transfer during conscious states
Source: eLife. 2024 Jan 5;13:e86547. doi: 10.7554/eLife.86547 (PMC10805384; doi:10.7554/eLife.86547)
Supplement: Supplementary file 3. [file elife-86547-supp3.pdf]

Table 1: Connectivity parameters for the waking state of the mean-field model of the basal ganglia-thalamo-cortical system, derived using Bayesian-genetic optimization.

	**Axonal range (r)**	**Damping rate (γ)**	**Synapto-dendritic Decay Rate (α)**	**Synapto-dendritic Rise Rate (β)**	**Axonal delay (τ)**	**Connection strength**
**Exc. $\rightarrow$ Exc.**	0.12278	220.0791	85.9279	322	0.0062323	0.0018327
**Inh. $\rightarrow$ Exc.**			42.555	281	0.04334	-0.0013089
**Relay $\rightarrow$ Exc.**			220.8828	193	0.028104	0.00071992
**Exc. $\rightarrow$ Inh.**	0.22666	232.1854	85.7514	175.0493	0.00037033	0.0011322
**Inh. $\rightarrow$ Inh.**			113.6299	620	0.033074	-0.00050632
**Relay $\rightarrow$ Inh.**			129.4099	555	0.025181	0.0003415
**GPe $\rightarrow$ Inh.**			75.6795	205.7867	0.034081	-0.0000168
**Exc. $\rightarrow$ TRN**	0.17182	73	45.089	492	0.036522	0.00029379
**Relay $\rightarrow$ TRN**			187	791	0.0022316	0.0000157
**GPe $\rightarrow$ TRN**			159.0547	153.6758	0.047298	-0.00032958
**Exc. $\rightarrow$ Relay**	0.218	137.9915	44.8479	483.8873	0.022911	0.00048973
**TRN $\rightarrow$ Relay**			182	129.5577	0.0017706	-0.00033052
**GPi $\rightarrow$ Relay**			162.841	347.9839	0.0014159	-0.000024
**GPe $\rightarrow$ Relay**			63.8256	914.7857	0.0090983	-0.0002165
**Exc. $\rightarrow$ D1**	0.1188	23	152.7024	258	0.00069557	0.00080197
**Relay $\rightarrow$ D1**			143.3016	521.6289	0.0015367	0.0000963
**D1 $\rightarrow$ D1**			122	262.8843	0.002218	-0.0001518
**GPe $\rightarrow$ D1**			78.6903	270.8312	0.03725	-0.0000887
**Exc. $\rightarrow$ D2**	0.071609	143	125	597.8874	0.00069498	0.00071134
**Relay $\rightarrow$ D2**			53.1836	390	0.002088	0.0000248
**D2 $\rightarrow$ D2**			74.6389	435.8297	0.029915	-0.00024613
**GPe $\rightarrow$ D2**			67.8765	294.734	0.045457	-0.00045438
**D1 $\rightarrow$ GPi**			116.0711	630.4158	0.0012356	-0.0000831
**GPe $\rightarrow$ GPi**			148.6143	403.3654	0.0006374	-0.0000316
**STN $\rightarrow$ GPi**			51.749	255.38	0.001192	0.00018528
**D2 $\rightarrow$ GPe**			23.4433	311	0.00061974	-0.00021758
**GPe $\rightarrow$ GPe**			79.2077	367.7875	0.0027569	-0.00010213
**STN $\rightarrow$ GPe**			127.4022	483.2848	0.00043817	0.00023124
**Exc. $\rightarrow$ STN**	0.086406	95	136	730	0.00074221	0.00010993
**GPe $\rightarrow$ STN**			118.5557	314	0.00059721	-0.0000276

Table 2: Population parameters for the waking state of the mean-field model of the basal ganglia-thalamo-cortical system, derived using Bayesian-genetic optimization.

	**Maximum firing rate (Q)**	**Firing threshold (θ)**	**Firing threshold spread (σ)**
**Cortex (excitatory)**	228.0316	0.029706	0.0012552
**Cortex (inhibitory)**	249	0.011629	0.0045276
**TRN**	751.9918	0.0013855	0.0029211
**Relay**	466	0.0034528	0.0043363
**D1**	97.0027	0.0056627	0.0039531
**D2**	13.5585	0.0084425	0.0031499
**GPi**	222.0355	0.0036668	0.0045221
**GPe**	202.0382	0.0047459	0.0027718
**STN**	255.7239	0.011212	0.0041919

Table 3: Connectivity parameters for the anesthesia state of the mean-field model of the basal ganglia-thalamo-cortical system, derived using genetic optimization.

	**Axonal range (r)**	**Damping rate (γ)**	**Synapto-dendritic Decay Rate (α)**	**Synapto-dendritic Rise Rate (β)**	**Axonal delay (τ)**	**Connection strength**
**Exc. → Exc.**	0.13371	365.323	153.1084	140.2695	0.0077477	0.001194
**Inh. → Exc.**			42.555	150.1111	0.060006	-0.0015235
**Relay → Exc.**			111.9569	223.3302	0.029002	0.00030845
**Exc. → Inh.**	0.24992	284.4327	134.7121	175.0493	0.00037033	0.0011322
**Inh. → Inh.**			115.707	901.3049	0.033074	-0.00050632
**Relay → Inh.**			129.4099	512.0002	0.036616	0.00030238
**GPe → Inh.**			121.6404	205.7867	0.043839	-1.68E-05
**Exc. → TRN**	0.17182	73	45.8769	466.2431	0.015442	0.00015174
**Relay → TRN**			41.5367	414.209	0.0012636	1.57E-05
**GPe → TRN**			312.1321	1146.8432	0.037738	-0.00032958
**Exc. → Relay**	0.14701	137.9915	42.8395	317.922	0.012198	0.00039605
**TRN → Relay**			36.5624	363.0627	0.00085722	-0.00033052
**GPi → Relay**			186.0943	158.6022	0.0014159	-2.40E-05
**GPe → Relay**			162.841	347.9839	0.0090983	-0.0002165
**Exc. → D1**	0.1188	17.0626	190.1072	131.2923	0.00069557	0.00078156
**Relay → D1**			54.443	166.2305	0.0014043	0.00010744
**D1 → D1**			66.048	773.6346	0.002218	-0.00013107
**GPe → D1**			130.4614	305.148	0.03725	-6.46E-05
**Exc. → D2**	0.071609	130.8712	53.4017	1381.7241	0.00069498	0.00071134
**Relay → D2**			67.268	829.9581	0.0017657	2.48E-05
**D2 → D2**			72.0903	390	0.029915	-0.00039284
**GPe → D2**			135.9065	665.8283	0.045457	-0.00036314
**D1 → GPi**			14.8752	725	0.0005457	-8.31E-05
**GPe → GPi**			54.7637	630.4158	0.00080621	-2.53E-05
**STN → GPi**			162.144	403.3654	0.0019464	5.48E-05
**D2 → GPe**			37	710	0.00040424	-0.00028375
**GPe → GPe**			23.4433	153.0925	0.0014851	-0.00022676
**STN → GPe**			185.906	549.1626	0.00043817	0.00025348
**Exc. → STN**	0.021519	52.1657	112.8993	659	0.0010297	0.0001557
**GPe → STN**			67.564	730	0.00053249	-2.76E-05

Table 4: Population parameters for the anesthesia state of the mean-field model of the basal ganglia-thalamo-cortical system, derived using genetic optimization.

	**Maximum firing rate (Q)**	**Firing threshold (θ)**	**Firing threshold spread (σ)**
**Cortex (excitatory)**	228.0316	0.054464	0.0011733
**Cortex (inhibitory)**	382.426	0.011629	0.0021614
**TRN**	1140.8187	0.0029636	0.0026014
**Relay**	466	0.0034528	0.0043363
**D1**	46.883	0.0072807	0.0063121
**D2**	13.5585	0.0084425	0.0037879
**GPi**	294.5833	0.0036668	0.0056883
**GPe**	131.961	0.0047459	0.0062872
**STN**	389.4177	0.011212	0.0041919

Table 5: Connectivity parameters for the generalized spike-and-wave seizure state of the mean-field model of the basal ganglia-thalamo-cortical system, derived using genetic optimization.

	**Axonal range (r)**	**Damping rate (γ)**	**Synapto-dendritic Decay Rate (α)**	**Synapto-dendritic Rise Rate (β)**	**Axonal delay (τ)**	**Connection strength**
**Exc. → Exc.**	0.12278	220.0791	85.9279	322	0.0062323	0.0018327
**Inh. → Exc.**			42.555	281	0.04334	-0.0013089
**Relay → Exc.**			220.8828	193	0.028104	0.00071992
**Exc. → Inh.**	0.22666	232.1854	85.7514	175.0493	0.00037033	0.0011322
**Inh. → Inh.**			113.6299	620	0.033074	-0.00050632
**Relay → Inh.**			129.4099	555	0.025181	0.0003415
**GPe → Inh.**			75.6795	205.7867	0.034081	-0.0000168
**Exc. → TRN**	0.17182	73	45.089	492	0.036522	0.00029379
**Relay → TRN**			187	791	0.0022316	0.0000157
**GPe → TRN**			159.0547	153.6758	0.047298	-0.00032958
**Exc. → Relay**	0.218	137.9915	44.8479	483.8873	0.022911	0.00048973
**TRN → Relay**			182	129.5577	0.0017706	-0.00033052
**GPi → Relay**			162.841	347.9839	0.0014159	-0.000024
**GPe → Relay**			63.8256	914.7857	0.0090983	-0.0002165
**Exc. → D1**	0.1188	23	152.7024	258	0.00069557	0.00080197
**Relay → D1**			143.3016	521.6289	0.0015367	0.0000963
**D1 → D1**			122	262.8843	0.002218	-0.0001518
**GPe → D1**			78.6903	270.8312	0.03725	-0.0000887
**Exc. → D2**	0.071609	143	125	597.8874	0.00069498	0.00071134
**Relay → D2**			53.1836	390	0.002088	0.0000248
**D2 → D2**			74.6389	435.8297	0.029915	-0.00024613
**GPe → D2**			67.8765	294.734	0.045457	-0.00045438
**D1 → GPi**			116.0711	630.4158	0.0012356	-0.0000831
**GPe → GPi**			148.6143	403.3654	0.0006374	-0.0000316
**STN → GPi**			51.749	255.38	0.001192	0.00018528
**D2 → GPe**			23.4433	311	0.00061974	-0.00021758
**GPe → GPe**			79.2077	367.7875	0.0027569	-0.00010213
**STN → GPe**			127.4022	483.2848	0.00043817	0.00023124
**Exc. → STN**	0.086406	95	136	730	0.00074221	0.00010993
**GPe → STN**			118.5557	314	0.00059721	-0.0000276

Table 6: Population parameters for the generalized spike-and-wave seizure state of the mean-field model of the basal ganglia-thalamo-cortical system, derived using genetic optimization.

	**Maximum firing rate (Q)**	**Firing threshold (θ)**	**Firing threshold spread (σ)**
**Cortex (excitatory)**	210.4443	0.041636	0.00052695
**Cortex (inhibitory)**	341.408	0.011629	0.0045276
**TRN**	841.0487	0.0020067	0.0029211
**Relay**	829.0906	0.0033459	0.0058172
**D1**	97.0027	0.004253	0.0058384
**D2**	24.9503	0.010996	0.0044716
**GPi**	222.0355	0.0022048	0.0059299
**GPe**	202.0382	0.0042228	0.0027718
**STN**	255.7239	0.011212	0.0041919
