# Supplementary material for: Criticality supports cross-frequency cortical-thalamic information transfer during conscious states
Source: eLife. 2024 Jan 5;13:e86547. doi: 10.7554/eLife.86547 (PMC10805384; doi:10.7554/eLife.86547)
Supplement: Supplementary file 4. [file elife-86547-supp4.pdf]

Table 1: We tested whether estimated differences in thalamocortical chaoticity across brain states were potentially driven by the spectral power of activity in the frequency range of interest (1-13 Hz). To do so, we used a permutation-based nonparametric ANCOVA with 10,000 permutations, following the method described in Supplementary File 1, Table 1. We set the median K-statistic across thalamic and cortical channels as the response variable, brain state (i.e. normal waking, generalized spike-and-wave seizure, anesthesia, or 5-MeO-DMT) as the group label, and set the covariate to the median relative spectral power between 1 and 13 Hz across all cortical and thalamic channels. We found that only brain state significantly explained the observed variance across subjects in the estimated chaoticity of slow thalamocortical electrodynamics, with no significant effect of spectral power or its interactions with brain state.

Variable	F-statistic	p-value
Brain state	62.7359	0.0001
Thalamocortical low frequency power	1.0899	0.2514
Brain state*low frequency power	2.9942	0.3063
