# Supplementary material for: Criticality supports cross-frequency cortical-thalamic information transfer during conscious states
Source: eLife. 2024 Jan 5;13:e86547. doi: 10.7554/eLife.86547 (PMC10805384; doi:10.7554/eLife.86547)
Supplement: Supplementary file 5. [file elife-86547-supp5.pdf]

Table 1: We here report the percentage of cortical channels, over all 10-second windows of data, which were classified as stochastic when bandpass filtered at the frequency range tested in the main body of the paper for chaoticity (1-13 Hz) (“Low-Frequency”) or lowpass filtered at the highest frequency range considered in our spectral information transfer analysis (104 Hz) (“Broadband”). Our stochasticity test was previously described in prior work (Toker et al., 2022). Note that “Altered State” refers to propofol anesthesia for the human ET patients and Long-Evans Rats, to generalized spike-and-wave seizures for the GAERS rats, and the psychedelic state for the C57BL/6 mice. Nearly all cortical channels in all subjects were classified as predominantly deterministic when applying the test to to low-frequency data, and a majority of cortical channels were classified as deterministic when applying the test to broadband data.

	Low-Frequency		Broadband	
	Normal Waking	Altered State	Normal Waking	Altered State
Human ET Patient 1	0.00	0.00	40.00	6.25
Human ET Patient 2	0.00	0.00	40.00	0.00
Human ET Patient 3	0.00	0.00	25.00	2.08
Human ET Patient 4	0.00	0.00	37.50	2.08
Human ET Patient 5	0.00	0.00	2.50	0.00
Human ET Patient 6	0.00	0.00	0.00	0.00
Human ET Patient 7	0.00	0.00	37.50	2.08
Human ET Patient 8	0.00	0.00	0.00	8.33
Human ET Patient 9	0.00	0.00	2.50	0.00
Human ET Patient 10	0.00	0.00	45.00	0.00
Long-Evans Rat 1	0.00	0.00	91.67	0.00
Long-Evans Rat 2	0.00	0.00	41.67	0.00
Long-Evans Rat 3	0.00	0.00	35.71	0.00
Long-Evans Rat 4	0.00	0.00	75.00	0.00
Long-Evans Rat 5	0.00	0.00	35.71	0.00
Long-Evans Rat 6	0.00	0.00	81.82	5.00
Long-Evans Rat 7	0.00	0.00	75.00	0.00
Long-Evans Rat 8	0.00	4.76	11.11	100.00
Long-Evans Rat 9	0.00	0.00	100.00	0.00
GAERS Rat 1	14.79	9.76	46.48	41.46
GAERS Rat 2	16.39	9.09	65.57	40.91
GAERS Rat 3	10.76	8.16	72.15	34.69
GAERS Rat 4	16.67	9.09	77.55	50.00
GAERS Rat 5	8.86	22.22	72.15	66.67
GAERS Rat 6	17.43	15.09	67.89	33.96
GAERS Rat 7	15.38	7.69	69.23	48.72
C57BL/6 Mouse 1	0.00	0.00	1.44	2.00
C57BL/6 Mouse 2	0.00	0.00	3.85	0.00
C57BL/6 Mouse 3	0.00	0.00	0.66	0.00
C57BL/6 Mouse 4	0.00	0.00	0.00	0.00
C57BL/6 Mouse 5	0.00	0.00	15.53	0.00
AVERAGE	3.23	2.77	40.97	14.33

Table 2: We here report the percentage of thalamic channels, over all 10-second windows of data, which were classified as stochastic when bandpass filtered at the frequency range tested in the main body of the paper for chaoticity (1-13 Hz) ("Low-Frequency") or lowpass filtered at the highest frequency range considered in our spectral information transfer analysis (104 Hz) ("Broadband"). As was the case for cortical channels (Table 1), nearly all thalamic channels in all subjects were classified as predominantly deterministic when the test was applied to low-frequency data, and a majority of thalamic channels were classified as deterministic when the test was applied to broadband data.

	Low-Frequency		Broadband	
	Normal Waking	Altered State	Normal Waking	Altered State
Human ET Patient 1	0	0	0	0
Human ET Patient 2	0	0	0	2.08
Human ET Patient 3	0	0	5.00	0
Human ET Patient 4	0	0	12.50	0
Human ET Patient 5	0	0	0	0
Human ET Patient 6	0	0	0	0
Human ET Patient 7	0	0	2.50	2.08
Human ET Patient 8	0	0	0	0
Human ET Patient 9	0	0	7.50	0
Human ET Patient 10	0	0	17.50	0
Long-Evans Rat 1	0	0	58.33	8.33
Long-Evans Rat 2	0	0	33.33	0
Long-Evans Rat 3	0	0	7.14	0
Long-Evans Rat 4	0	0	50.00	22.22
Long-Evans Rat 5	7.14	10.00	28.57	10.00
Long-Evans Rat 6	0	0	72.73	0
Long-Evans Rat 7	0	0	58.33	9.09
Long-Evans Rat 8	0	0	88.89	0
Long-Evans Rat 9	0	6.25	0	81.25
GAERS Rat 1	14.08	21.95	69.01	100.00
GAERS Rat 2	14.76	31.82	70.49	86.36
GAERS Rat 3	13.61	28.57	76.58	95.92
GAERS Rat 4	12.93	18.18	71.09	100.00
GAERS Rat 5	12.03	0	72.78	88.89
GAERS Rat 6	15.60	15.09	68.81	77.36
GAERS Rat 7	16.03	25.64	80.13	76.92
C57BL/6 Mouse 1	0	0	56.25	41.33
C57BL/6 Mouse 2	0	0	61.54	4.64
C57BL/6 Mouse 3	0	0	54.61	42.97
C57BL/6 Mouse 4	0	0	34.87	6.12
C57BL/6 Mouse 5	0	0	45.96	25.85
AVERAGE	3.79	5.63	42.84	31.40

Table 3: To ensure that our classifications of the majority of empirical channels as deterministic was not an artifact induced by our bandass or lowpass filtering, we repeated the same analysis on pure noise with different colors or spectral exponents. As with our empirical recordings, we here report the percentage of noise samples (out of 100) that were classsified as predominantly stochastic. Although the majority of noise samples were mis-classified as predominantly deterministic when bandpass filtered between 1-13 Hz ("Low-frequency"), the average percentage of classifications of stochasticity in this frequency range (15%) was 4.37 times higher than the averages reported for our empirical channels (Supplementary File 5, Tables 1-2). Likewise, a large majority of noise samples were classified as stochastic when lowpass filtered at 104 Hz ("Broadband"), whereas a majority of empirical channels were classified as deterministic when considering this frequency range (Supplementary File 5, Tables 1-2). Overall, then, these results suggest that the empirical neural electrodynamics analyzed in this paper are predominantly deterministic. This validates the use of the 0-1 chaos test (which assumes that a signal is predominantly deterministic) in evaluating varying levels of chaotcity in the electrodynamics of the brain.

	Low-frequency	Broadband
White Noise	17	97
Pink Noise	18	85
Red Noise	11	87
